# Mild parkinsonian signs and incidence of Motoric Cognitive Risk syndrome

**DOI:** 10.1002/alz.089720

**Published:** 2025-01-03

**Authors:** Victoire Leroy, Ayers Emmeline, Joe Verghese

**Affiliations:** ^1^ Tours University Hospital, Tours France; ^2^ Department of Neurology, Division of Cognitive and Motor Aging, Albert Einstein College of Medicine, New York, NY USA; ^3^ Department of neurology, Division of Cognitive and Motor Aging, Albert Einstein College of Medicine, New York, NY USA

## Abstract

**Background:**

Mild parkinsonian signs (MPS) are prevalent in older adults and linked to an increased risk of dementia. However, their association with Motoric Cognitive Risk syndrome (MCR), a pre‐dementia syndrome characterized by slow gait speed and cognitive complaints, is unclear. This study aims to examine the association of MPS with incident MCR.

**Method:**

Non‐demented older adults without Parkinson’s disease were monitored annually (average follow‐up 30 months). We excluded participants with MCR at baseline and dementia incidence without previous MCR, as non‐cases. MPS were defined at baseline by the presence of bradykinesia, rigidity, or rest tremor rated by a study clinician using the Unified Parkinson’s Disease Rating Scale (UPDRS). MCR was diagnosed using established criteria at baseline and follow‐up. The association of baseline MPS and incident MCR was examined using survival analysis and reported as hazard ratio and 95% confidence intervals (CI).

**Result:**

Of the 580 participants, 227 (39.1%) had MPS and 44 (7.5%) had MCR at baseline. Of the 513 initially MCR‐free participants, 46 (8.9%) developed incident MCR. Incident MCR cases had a higher prevalence of MPS at baseline compared to participants who did not develop MCR (OR adjusted for age, geriatric depression scale (GDS) score, and stroke history = 3.8, 95% CI 1.8‐8.2), particularly bradykinesia (52.3% vs. 19.0%; p<0.001) and rigidity (54.5% vs. 28.1%; p<0.001). They were older (p<0.001), more disabled (p<0.001), and had more strokes (p = 0.005), and depressive symptoms (p<0.001) compared to individuals who did not develop MCR. Participants with MPS at baseline had a greater risk of developing incident MCR (HR adjusted for age, GDS score, and stroke history = 3.7, 95% CI 1.8‐7.7).

**Conclusion:**

MPS predicted the onset of MCR, suggesting a significant role in the pathogenesis and in improving early assessment of risk for MCR.

